# Time-of-Travel Methods for Measuring Optical Flow on Board a Micro Flying Robot

**DOI:** 10.3390/s17030571

**Published:** 2017-03-11

**Authors:** Erik Vanhoutte, Stefano Mafrica, Franck Ruffier, Reinoud J. Bootsma, Julien Serres

**Affiliations:** Aix-Marseille Université, CNRS, ISM UMR7287, 13288 Marseille Cedex 09, France; erik.vanhoutte@univ-amu.fr (E.V.); stefano.mafrica@mpsa.com (S.M.); franck.ruffier@univ-amu.fr (F.R.); reinoud.bootsma@univ-amu.fr (R.J.B.)

**Keywords:** optic flow sensor, sense and avoid, VLSI retina, micro air vehicle (MAV), bionics, bio-inspired robotics, biorobotics

## Abstract

For use in autonomous micro air vehicles, visual sensors must not only be small, lightweight and insensitive to light variations; on-board autopilots also require fast and accurate optical flow measurements over a wide range of speeds. Using an auto-adaptive bio-inspired Michaelis–Menten Auto-adaptive Pixel (M${}^{2}$APix) analog silicon retina, in this article, we present comparative tests of two optical flow calculation algorithms operating under lighting conditions from $6\times 10^{-7}$ to $1.6\times 10^{-2}$ W·cm${}^{-2}$ (i.e., from 0.2 to 12,000 lux for human vision). Contrast “time of travel” between two adjacent light-sensitive pixels was determined by thresholding and by cross-correlating the two pixels’ signals, with measurement frequency up to 5 kHz for the 10 local motion sensors of the M${}^{2}$APix sensor. While both algorithms adequately measured optical flow between 25 ${}^{\circ }$/s and 1000 ${}^{\circ }$/s, thresholding gave rise to a lower precision, especially due to a larger number of outliers at higher speeds. Compared to thresholding, cross-correlation also allowed for a higher rate of optical flow output (99 Hz and 1195 Hz, respectively) but required substantially more computational resources.

## 1. Introduction

To detect and avoid obstacles in unpredictable environments, flying insects rely heavily on optical flow (OF) [1], defined as the vector field of angular velocities of contrasted points, edges or surfaces resulting from the relative motion between the observer and the surrounding objects [2,3]. Their OF-based strategies therefore provide inspiration for the development of smart autopilots for micro-air-vehicles (MAVs) [1,4,5] and smart artificial retinas [6,7,8,9,10,11,12].

Insect-sized MAVs are increasingly becoming a reality [13,14,15,16,17,18] and have to be fitted in the future with sensors and flight control devices enabling them to perform all kinds of aerial maneuvers, including ground and obstacle avoidance, terrain-following and landing.

The fitted sensors should not only be non-emissive to allow an MAV to save energy resources and to be stealth in flight, but must also guarantee a high refresh rate because GPS (GPS stands for the Global Positioning System) signals are limited in both spatial (∼1 m) and temporal resolution (∼7 Hz). Micro cameras have been proposed for MAV applications (e.g., a PAL (PAL stands for Phase Alternating Line) -camera with 720 × 576 pixels at 25 fps [16] or a CMOS (CMOS stands for Complementary Metal-Oxide Semiconductor) camera with 752 × 480 pixels at 80 fps [19]), including visual-based simultaneous localization and mapping algorithms (SLAM).

A few cameras deliver fast and reliable OF measurement, but still remain bulky: (i) a PIX4FLOW CMOS camera with 4× binning and 188 × 120 pixels delivers only one OF vector—direction and magnitude—at 250 fps [20]; and (ii) the event-based camera called DAVIS (DAVIS stands for Dynamic and Active-pixel VIsion Sensor) with 240 × 180 pixels delivers accurate OF measurement asynchronously [21,22]. The drawbacks of CMOS cameras moreover include their poor robustness to various lighting conditions (e.g., ∼600 lux in [20]) and their high (pixel number based) demands on computational resources.

It is essential for MAV applications to use lightweight, low-power consumption and high refresh rate sensors to comply with fast dynamics and to stay reactive in unpredictable environments. Hovering requires lower refresh rates than obstacle avoidance maneuvers while flying at high speed. Inertial measurement units (IMUs) are used to stabilize an MAV in flight or simply to hover at a refresh rate up to 500 Hz. Recently, both an IMU sampled at 500 Hz and OF measurements sampled at 25 Hz were fused using an extended Kalman filter to make an MAV hover robustly [23]. The authors’ idea was to use the OF direction, but not its scale to correct for inertial sensor drift in particular during changes of direction and to enhance the accuracy of a hovering positioning: this strategy helps in updating both an attitude and a positioning control outputs at 100 Hz [23]. Lately, a 40-gram pocket drone avoided obstacles on the basis of OF computed at 20 Hz [24]. The refresh rate of the visual processing appears to be the key parameter to make an MAV achieve “aggressive” maneuvers based on on-board vision (as demonstrated in [25]) with sufficient reactive abilities in unpredictable environments. More generally, the criteria for the evaluation of the potential of OF sensors for MAV applications include:

- robustness to light level variations, defined by the number of irradiance decades in which the visual sensor can operate;
- range of OF angular speeds (or magnitudes) covered, defined by the minimum and maximum values measured;
- accuracy and precision, defined by systematic errors and coefficients of variation;
- output refresh rate, defined by the instantaneous output frequency.

A recent OF sensor was based on the M${}^{2}$APix (M${}^{2}$APix stands for Michaelis–Menten auto-adaptive pixel) retina that can auto-adapt in a seven-decade lighting range and responds appropriately to step changes up to ±3 decades [26]. The pixels do not saturate thanks to the (normalization based) intrinsic properties of the Michaelis–Menten equation [27]. A comparison of the characteristics of auto-adaptive Michaelis–Menten and Delbrück pixels under identical lighting conditions demonstrated better performance of the Michaelis–Menten pixels in terms of dynamic sensitivity and minimum contrast detection [12].

A single local motion sensor (LMS) fitted with two auto-adaptive pixels has been demonstrated [7] to allow measuring an OF range from 50 to 350 °/s at relatively constant output refresh rate (∼5–7 Hz) despite variations in lighting conditions from ∼50 lux to 10,000 lux. With a larger number of LMSs, output refresh rate increased (64 Hz with 5 LMSs) in an OF range from 25 to 350 °/s (i.e., 1.1-decade) at a constant natural lighting ∼1500 lux [8]. A similar OF range from 50 to 250 °/s (i.e., 0.7-decade) was measured with a semi-panoramic artificial eye, called CurvACE [9,28]. The OF range can also be adjusted by tuning the inter-pixel angle, as done for outdoor flights in [29]. An ad hoc interpolation-based “time-of-travel” algorithm relying on thresholding was embedded into a small 40 MIPS (MIPS stands for Million Instructions Per Second) dsPIC${}^{®}$ (Microchip Technology Inc., Chandler, AZ, USA) microcontroller to allow a trade-off between OF range [25 °/s; 350 °/s], accuracy ($\sigma_{error}$ < 15 °/s), the sample rate of the visual signals (200 Hz), computational resources (10.5% on average of the processing time available) and the OF refresh rate (95 Hz with 5 LMSs) [30]. With this limited OF range, the robot’s operating OF was adjusted to the middle of the range: 125 °/s in [31], 160 °/s in [32], or even 200 °/s in [33] (which was by the way quite high in comparison with related works: ∼60–70 °/s in [17,34,35,36]).

In this paper, we compare two different methods for the calculation of OF. Both methods are based on time-of-travel algorithms, relying either on thresholding or on cross-correlation of adjacent bandpass-filtered visual signals. The two algorithms are applied on the same visual signals provided by the M${}^{2}$APix retina when operating under different combinations of the following conditions:

- in an OF range from 25 °/s to 1000 °/s;
- under irradiance conditions varying from 6 $\times \;10^{-7}\;$W·cm${}^{-2}$ to 1.6 $\times \;10^{-2}\;$W·cm${}^{-2}$;
- with sampling rates between 100 Hz and 1 kHz;
- in real flight when fitted onto a 350-gram MAV.

It will be shown that at low sampling rate ($F_{e}<500$ Hz), the cross-correlation method is more precise than the thresholding method over a wide range of OF speeds. Requiring much less computational resources, the thresholding method functions adequately at a high sampling rate ($F_{e}$ close to 1 kHz), with the OF range covered depending mainly on the inter pixel angle Δφ.

In Section 2, optics and front-end pixels of the M${}^{2}$APix sensor will be briefly introduced with respect to previous work based on the M${}^{2}$APix sensor. In Section 3, the two methods of OF computation based on time-of-travel algorithms will be described in detail. In Section 4, both time-of-travel algorithms applied on the same M${}^{2}$APix sensor visual signals will be tested in an OF range from 25 °/s to 175 °/s at different levels of lighting, then in an OF range from 25 °/s to 1000 °/s at a constant lighting by using an experimental setup based on a moving pattern. In Section 5, both time-of-travel algorithms with the M${}^{2}$APix sensor will be tested on-board a 350-gram quadrotor during flights over a flat ground textured with a natural scene. Both time-of-travel methods will be compared in terms of computational resources, accuracy, precision and output refresh rate. In Section 6, the benefits of each method will be discussed, leading to the conclusion that time-of-travel algorithms with a M${}^{2}$APix sensor can measure a wide OF range at high refresh output frequency despite large lighting variations in real flight.

## 2. Optics and Front-End Pixels of the M${}^{2}$APix Sensor

**Figure 1:**
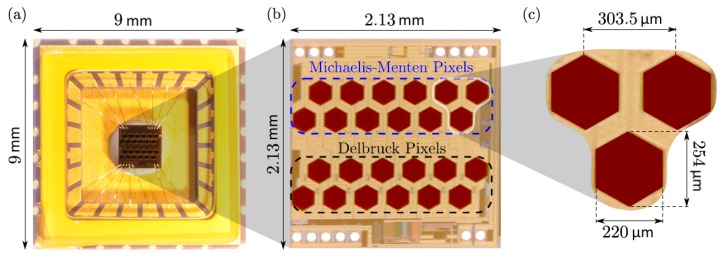
(**a**) The M${}^{2}$APix sensor chip with its wire bounding; (**b**) the silicon retina composed of 12 Michaelis–Menten pixels and 12 additional Delbrück pixels; (**c**) view of three neighboring pixels. From [12].

The M${}^{2}$APix sensor is composed of two groups of light-sensitive pixels: 12 Michaelis–Menten pixels and 12 additional Delbrück pixels [12] (Figure 1a,b). Each group is composed of two rows of six pixels with rows offset by half the inter-pixel distance (Figure 1c). In this paper, we only used the 12 Michaelis–Menten pixels, sampled at the frequency $F_{e}$ (from 100 Hz to 1 kHz, depending on the experimental setting) by an Overo AirSTORM computer-on-module (COM) (Gumstix, Redwood City, CA, USA) featuring a 1-GHz CPU DM3703 processor (Texas Instruments, Dallas, TX, USA) comprising an ARM Cortex-A8 architecture.

The M${}^{2}$APix sensor is equipped with a lens taken from a Raspberry Pi camera (focal length = 2 mm). The lens is deliberately defocused (distance between the lens and the retina = 1 mm) to provide a bio-inspired low-pass spatial filter [37]. The optical configuration can be described by the inter-pixel angle Δφ = $4.3^{\circ }$ and the full width at half height of the Gaussian sensitivity, the acceptance angle Δρ $=\;3.0^{\circ }$, thereby respecting the $\frac{\Delta \rho }{\Delta \varphi }<2$ rule for proper visual sampling established for diurnal insects. In this configuration, the M${}^{2}$APix sensor has a field of view of $32.8^{\circ }\times 13.4^{\circ }$ in the horizontal and vertical plane, respectively.

In most animals’ visual systems, the photoreceptors have been observed to auto-adapt their response to the ambient light level [27]. The M${}^{2}$APix pixels are also auto-adaptive, as their responses are normalized by a signal that depends on the low-pass filtered average of all of the 12 photoreceptors’ currents, according to the Michaelis–Menten equation [12,38]. The adaptation time constant of the pixels’ response was set here at 0.5s by using an external capacitor of 47 nF (see [12] for more details). This allows the amplitude of the output signals to remain within the same constant range notwithstanding variations in light level and, therefore, makes it possible for the pixels to operate in high-dynamic-range lighting conditions.

## 3. Optical Flow Computed by Time-Of-Travel-Based Algorithms

Time-of-travel algorithms compute the magnitude of OF (Equation (1)) by measuring the time delay Δt between the signals of two adjacent photoreceptors (here Michaelis–Menten pixels) constituting a local motion sensor (LMS):

(1) $$OF=\frac{\Delta \varphi }{\Delta t}$$

**Figure 2:**
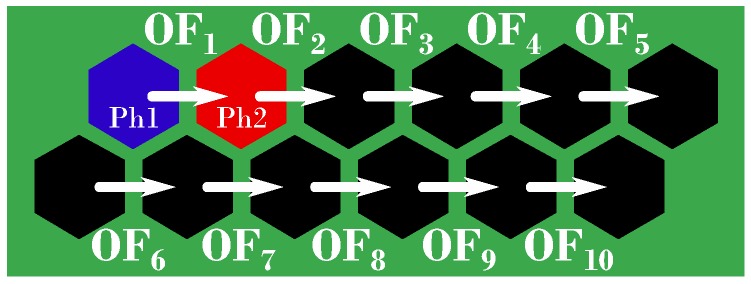
The 12 Michaelis–Menten pixels (Figure 1b) and the 10 corresponding local motion sensors (LMSs). LMSs are unidirectional and can only measure the OF magnitudes $OF_{i}$ i∈{1,...,10} in 1D. The optics geometry of the first two-pixel LMS (composed of the red and blue pixels) is described in Figure 3a.

In the work presented here, we used 10 LMSs (two rows of five), each computing the 1D OF in the direction parallel to the rows of pixels, as shown in Figure 2.

### 3.1. Time-Of-Travel Based on Signal Thresholding

This time-of-travel algorithm is based on opto-electrophysiological studies of the fly’s lobula plate tangential cells (H1-neuron) [39] and is described in detail in [4,7,8,29].

**Figure 3:**
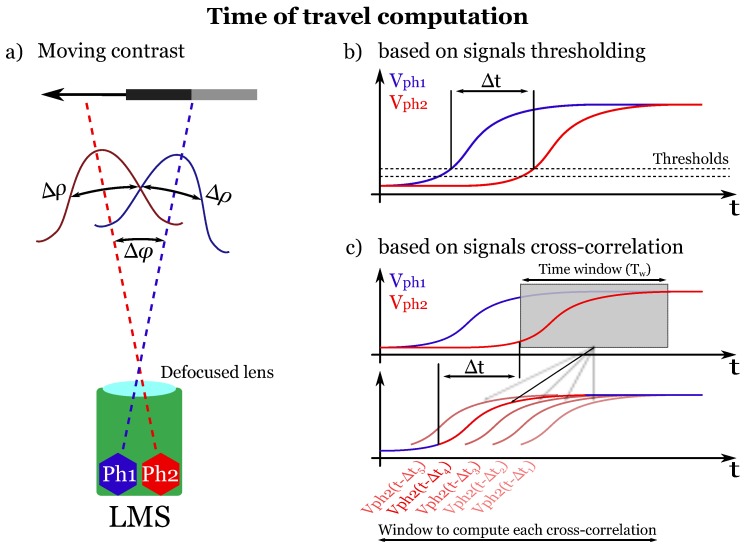
(**a**) Optics geometry of a LMS. A defocused lens confers an inter-pixel angle Δφ between the optical axes of two adjacent pixels Ph1 (blue) and Ph2 (red) and an acceptance angle Δρ given by the width of the Gaussian angular sensitivity at half height. The output signals $V_{ph1}$ and $V_{ph2}$ coming from pixels Ph1 and Ph2, respectively, are naturally delayed by a “time-of-travel” Δt when a contrast is moving in front of them (Figure 3b,c). The OF can therefore be computed with Equation (1). (**b**) Principle of the time-of-travel algorithm based on signal thresholding: the time delay Δt is computed when both signals reach a given hysteresis threshold [4,7,8,29]. (**c**) Principle of the time-of-travel algorithm based on signals’ cross-correlation: the time delay Δt is computed as the time delay giving the maximum cross-correlation coefficient *ϱ* between the delayed and non-delayed signal (see Figure 2 in [26] for details). Note that for simplicity, the time-of-travel computation is shown here using the raw signals $V_{ph1}$ and $V_{ph2}$, but it is actually computed after band-pass filtering these signals (see [4,7,8,26,29] for details).

The algorithm is illustrated in Figure 3b and computes the “time-of-travel” Δt by means of signal thresholding. To this end, the output of each pixel is first digitally band-pass filtered with cut-off frequencies of 3 Hz and 30 Hz. Two adjacent filtered signals are then thresholded (hysteresis thresholding) in order to measure the time delay Δt between them: a counter is started when the first pixel’s signal (the blue signal in Figure 3b) reaches a threshold, and it is stopped when the second pixel’s signal (the red signal in Figure 3b) reaches the same threshold. Lastly, the OF magnitude is computed using Equation (1).

The key parameter in this algorithm is the threshold value. A too large threshold value will allow detection only of high-amplitude pixel signals induced by strong contrasts and is therefore likely to neglect relevant signals. A too small threshold value will allow noise to cross the threshold and induce erroneous OF values. The thresholds’ values (hysteresis thresholding in Figure 3b) were therefore adjusted manually with respect to the best trade-off between the maximum refresh rate of the LMSs’ output and the minimum number of outliers.

In the experiments presented here, the high threshold was set at 5% of maximum signal amplitude (half the value of the high threshold for the low threshold for the hysteresis comparator; see Figure 3b), and all time delays falling outside a specific range (Δt from 4 to 717 ms, i.e., OF from 6 to 1145 ${}^{\circ }$/s) were excluded and considered as outliers. The algorithm was run at the pixels’ sampling frequency $F_{e}$, which varied between 100 Hz and 1 kHz, giving a quantization time $T_{e}=\frac{1}{F_{e}}$ varying between 1 ms and 10 ms. The OF was then computed by using Equation (1), where Δt is a multiple of $T_{e}$. The OF resolution ΔOF of this method is therefore non-constant, as it is given by an inverse function of the time delay Δt.

### 3.2. Time-of-Travel Based on Signals’ Cross-Correlation

This algorithm was inspired by the correlation-based models [40,41] and is based on signals’ cross-correlation (Figure 3c), as presented in [26]. First, the pixels’ output signals are digitally filtered with the same band-pass filter used in the thresholding algorithm (see Section 3.1). Then, one of the two signals in each pair of adjacent pixels is delayed by several time delays $\Delta t_{i}$, and the Pearson cross-correlation coefficients $\varrho_{i}$ are computed between the delayed ($V_{ph2}$ in Figure 3c) and non-delayed signals ($V_{ph1}$ in Figure 3c) within a fixed time window $T_{w}$. Lastly, the time delay $\Delta t_{m}$ giving the maximum coefficient $\varrho_{m}$ is obtained and used to compute the OF using Equation (1) as long as $\varrho_{m}$ is greater than a given threshold $\varrho_{thr}$. This threshold on the cross-correlation coefficients was set at 0.99 to avoid OF measurement errors due to signals mismatching [26].

The size of the fixed time window $T_{w}$ is one of the key parameters and requires being tuned as function of the signals’ bandwidth in order to have reliable cross-correlation coefficients ($\varrho_{i}$). Since the signals’ bandwidth is given by the band-pass filter (i.e. 3–30 Hz; see Section 3.1), $T_{w}$ was fixed here at a value of $T_{w}=0.14\;s$ so that $3\leq \frac{1}{T_{w}}\leq 30$. The cross-correlation time window was also defined as $T_{w}=N\cdot T_{e}$, with *N* being the number of samples in the time window. Therefore, a sampling rate $F_{e}=$ 500 Hz entails N=70, $F_{e}=$ 250 Hz entails N=35, and so on.

To compute the cross-correlation coefficients $\varrho_{i}$, the signal time window $T_{w}$ can be delayed by time steps $\Delta t_{i}$ that are calculated from the desired OF values $OF_{i}$ using the inverse function given in Equation (1). This can be done by taking time steps $\Delta t_{i}$ between two consecutive sampling steps ($n\;T_{e}$ and $\left(n+1\right)\;T_{e}$) after linearly interpolating the sampled signals. Therefore, by choosing the desired OF values $OF_{i}$ using constant steps, the computed OF will as a result be quantized with a constant resolution ΔOF, contrary to the algorithm based on signal thresholding (see Section 3.1).

## 4. Measuring Optical Flow with a Moving Texture

### 4.1. Method

**Figure 4:**
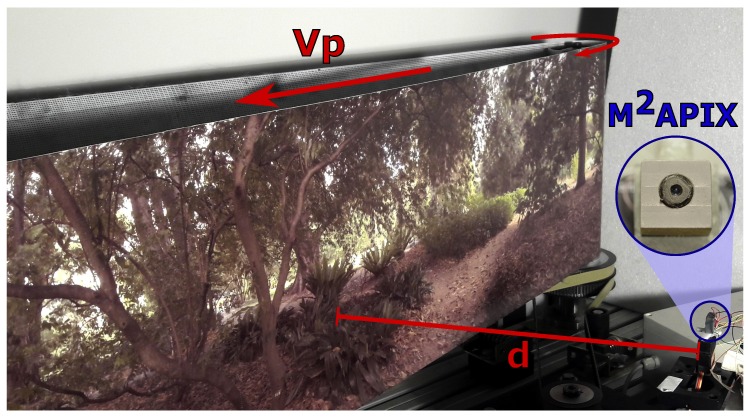
Experimental setup using a moving pattern and varying the artificial lighting to test algorithms with the M${}^{2}$APix sensor both in various OF ranges and in various lighting conditions. The pattern speed $V_{p}$ varied from 0 to 1.4 m·s${}^{-1}$; the sensor was placed perpendicularly in front of the moving pattern at a distance *d* from 8 cm to 40 cm.

In the following experiments, the M${}^{2}$APix sensor was fixed perpendicularly to a moving pattern whose linear speed could be controlled (Figure 4). Lighting was provided by three sources: daylight coming through the top windows (intensity manipulated by means of blinds), fluorescent tubes attached to the ceiling (intensity manipulated by means of a dimmer) and an LED projector that could be positioned in front of the moving pattern for high luminosity conditions. The M${}^{2}$APix sensor was thus confronted with a pure translational wide-range OF (from 25°/s to 1000°/s) under various lighting conditions (from 6 $\times \;10^{-7}\;$W·cm${}^{-2}$ to 1.6 $\times \;10^{-2}$W·cm${}^{-2}$, i.e., from 0.2 lux to 12,000 lux for human vision).

The moving-pattern experiments were separated in two parts: in the first part, the sensor was placed at a distance *d* = 40 cm from the moving pattern, and both the pattern speed and the lighting conditions were varied, whereas in the second part, only the pattern speed was varied at long constant steps, and the sensor was placed closer to the moving pattern (*d* = 8 cm) to obtain high OF.

**Figure 5:**
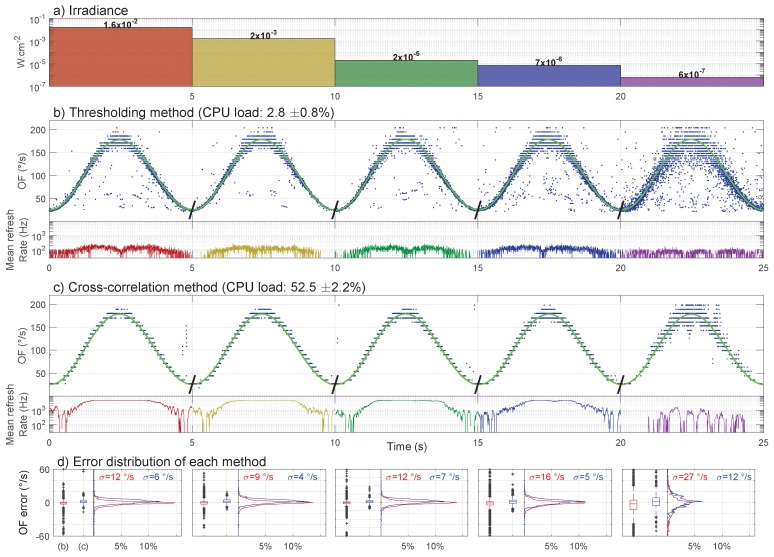
(**a**) The five levels of irradiance from 6 $\times \;10^{-7}\;$W·cm${}^{-2}$ to 1.6 $\times \;10^{-2}\;$W·cm${}^{-2}$ (i.e., from 0.2 lux to 12,000 lux for human vision) used during the first part of the experiments (see the experimental setup shown in Figure 4). (**b**,**c**) OF measurements obtained with the M${}^{2}$APix sensor using (b) the thresholding method ($F_{e}$ = 1 kHz) and (c) the cross-correlation method ($F_{e}$ = 500 Hz) for the 10 LMSs during 10 laps of the moving pattern. Tests were run separately for each of the five lighting conditions. Each blue dot represents one OF measurement of one LMS; however, the mean output refresh rate is the average of the number of OF measurement from the 10 LMSs in 1 s. The green line represents the OF ground-truth computed using Equation (2). (**d**) Error distribution of each time-of-travel method: the thresholding method is in red and the cross-correlation method in blue.

During the first part, each of the two algorithms was tested at five different irradiance levels. For each irradiance level, the pattern angular speed $OF_{pattern}$ varied over 10 periods as a sinus function with an angular frequency f=0.2πrad/s and a magnitude speed $V_{p}$ varying from $V_{pmin}$ = 0.18 m·s${}^{-1}$ to $V_{pmax}$ = 1.22 m·s${}^{-1}$ (Equation (2)). With the M${}^{2}$APix sensor placed at a distance *d* = 40 cm (Figure 4), the moving pattern generated an OF range from 25°/s–175°/s (green lines in Figure 5b,c), according to the following equation:

(2) $$OF_{pattern}=\frac{180}{\pi }\left(\frac{V_{pmax}-V_{pmin}}{2d}\cdot \cos\left(2\pi ft+\pi \right)+\frac{V_{pmax}+V_{pmin}}{2d}\right)$$

During the second part, the level of lighting was fixed at an irradiance of 7 ×$10^{-4}$ W·cm${}^{-2}$, and the two algorithms were used to compute OF in a wide OF range, from 25 °/s to 1000 °/s, by varying both the pattern speed ($V_{p}$ from 0 to 1.4 m·s${}^{-1}$) and the sensor’s distance *d* (see Figure 4). In particular, to obtain an OF ranging from 25 °/s to 200 °/s, *d* was set at 40 cm, and $V_{p}$ varied from 0.18 m·s${}^{-1}$ to 1.34 m·s${}^{-1}$; whereas, for an OF ranging from 250 °/s to 1000 °/s, *d* was set at 8 cm, and $V_{p}$ varied from 0.35 m·s${}^{-1}$ to 1.4 m·s${}^{-1}$.

These two experiments allowed us to compare the precision and the output refresh rate of both time-of-travel algorithms when using an auto-adaptive retina, such as the M${}^{2}$APix sensor in a wide light-level range (five-decade) and in a wide OF range (1.6-decade). However, the results should be similar with any other auto-adaptive retina implementing a similar auto-adaptation process.

The two algorithms were run at the highest operating rate/frequency that still guaranteed a proper operation for future embedded MAV applications, taking into account the constraint to maintain 40% of CPU (CPU stands for central processing unit) load-free for MAV control and navigation tasks. Therefore, the time-of-travel algorithm based on signals thresholding was run at 1000 Hz, whereas the time-of-travel algorithm based on signals cross-correlation was run at 500 Hz due to the high CPU load of this method.

### 4.2. Results

Figure 5 shows the OF measurements and their instantaneous refresh rates obtained when using the thresholding method (Figure 5b) and the cross-correlation method (Figure 5c) at five different light levels (five colored columns in Figure 5a–c). The OF measurements corresponding to the five light levels shown in Figure 5a were actually obtained during five distinct tests (see the oblique black line segments separating each test at 5, 10, 15 and 20 s in Figure 5b,c). Furthermore, for each light level (each column/sinusoidal period in Figure 5b,c), the OF measurements of the 10 LMSs obtained during 10 sinusoidal periods of the moving pattern (see Section 4.1) were overlaid on only one sinusoidal period in order to show all of the measurements in one figure. The refresh rates given in the lower plots in Figure 5b,c were computed by low-pass filtering the average number of OF measurements obtained at every time step over the last three and the next three steps (six centered point low-pass digital averaging filter). The OF errors were computed as the difference between the OF measurements and their ground-truth values (blue dots and green lines in Figure 5b,c, respectively). The boxplots and the distributions of these errors shown in Figure 5d were computed with 1 °/s beams in the [150 °/s; 150 °/s] range.

The number of outliers is much lower for the cross-correlation method (Figure 5c) than for the thresholding method (Figure 5b). While both error distributions (Figure 5d) have a similar standard deviation, the boxplots reveal the differences in the number of outliers.

The thresholding method running at 1 kHz (Figure 5b) is very light in terms of CPU load (2.8%±0.8%) and provides a good OF accuracy and precision until an irradiance of 7 $\times \;10^{-6}\;$W·cm${}^{-2}$ (blue column in Figure 5) despite more outliers than the cross-correlation method. However, the refresh rate obtained is relatively low (average of 97 Hz and maximum of 607 Hz for the full sensor). At very low irradiance, i.e., 6 $\times \;10^{-7}\;$W·cm${}^{-2}$, both the OF precision and the refresh rate are strongly deteriorated (see the violet column in Figure 5).

In contrast, the cross-correlation method running at 500 Hz gives a much lower number of outliers (boxplots in Figure 5d) and a much higher refresh rate (average of 2326 Hz and maximum of 5000 Hz for the full sensor), but it is very heavy in terms of CPU load (52.5%±2.2%). Similarly to the thresholding method, at very low light levels, i.e., 6 $\times \;10^{-7}\;$W·cm${}^{-2}$ (violet column in Figure 5), the refresh rate is deteriorated, especially at very low OF values. With the cross-correlation method, the OF precision is however less deteriorated than with the thresholding method.

The cross-correlation method does not delay signals in constant steps, but with steps following an inverse function to obtain a constant OF-measurement resolution.

**Figure 6:**
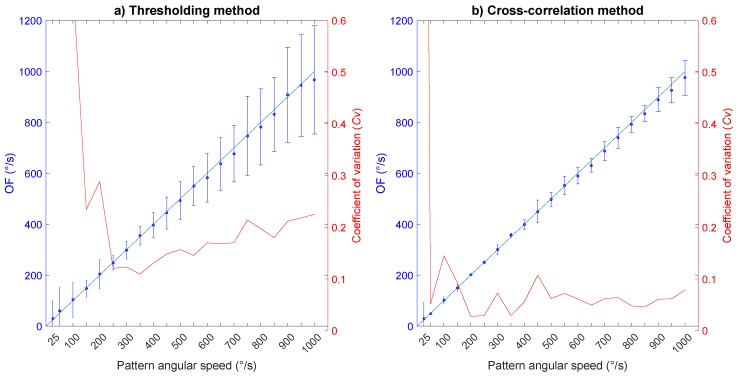
Accuracy of the M${}^{2}$APix sensor’s output when using: (**a**) the time-of-travel algorithm based on signal thresholding with $F_{e}$ = 1 kHz; and (**b**) the time-of-travel algorithm based on signal cross-correlation with $F_{e}$ = 500 Hz. The tests were performed at a constant level of irradiance (7$\times 10^{-4}$ W·cm${}^{-2}$), and the OF produced by the moving pattern was made to vary from 25 °/s to 1000 °/s with steps of 50 °/s (except for the first step). Each graph shows the average *μ* (blue circles), the standard deviation *σ* (blue vertical bars) and the coefficient of variation $\frac{\sigma }{\mu }$ (red lines) of the OF measurements with respect to the angular speed of the moving pattern (see Figure 4). The ground-truth values of the OF are given by the blue lines.

In the second experiment, various levels of OF were tested from 25 °/s to 1000 °/s with an OF step of 50 °/s (except for the first step) at a constant irradiance level (7 $\times \;10^{-4}$ W·cm${}^{-2}$). For each step, the OF mean *μ*, standard deviation *σ* and coefficient of variation $\frac{\sigma }{\mu }$ were computed for both time-of-travel algorithms, as shown in Figure 6.

Inspection of Figure 6 reveals that the OF mean values *μ* are very close to the ground-truth values for both methods (blue circles in Figure 6). However, the standard deviation *σ* (blue vertical bars in Figure 6), and therefore, the coefficient of variation $C_{V}=\frac{\sigma }{\mu }$ (red curves in Figure 6), is higher when using the thresholding method ($C_{V}>0.1$ in Figure 6a) than the cross-correlation method ($C_{V}<0.1$ in Figure 6b), except for very low OF values, i.e., 25°/s, where the precision is nearly the same.

The increase in the coefficient of variation with the thresholding method at low angular speed could be explained by the auto-adaptive response of the M${}^{2}$APix sensor that respects a time constant of 0.5 s on average on the whole sensor. Consequently, the lower the angular speed, the higher the number of outliers, as the pixels’ adaptation time constant is close to the signals’ dynamics, therefore generating matching errors. If one of the two signals does not reach the hysteresis threshold, no OF is measured. This does not happen with the cross-correlation method because the OF is computed by considering a time window comprising *N* samples (see Section 3.2).

Nevertheless, even with the cross-correlation method, the coefficient of variation increases abruptly around 25°/s. If signals coming from the same contrast on two adjacent pixels are too different due to the auto-adaptation process, the minimum cross-correlation coefficient (set at $\varrho_{i}=0.99$) will not be reached, and no OF measurement will be generated. Moreover, at low speed, the signal dynamics is close to the fixed time window size set at $T_{w}=0.14$ s. Different signals may therefore appear similar in this time window, thereby generating matching errors.

Toward the high angular speeds, the delay Δt between each photoreceptors’ signal approaches the M${}^{2}$APix sensor sampling rate $F_{e}$. The OF calculation therefore becomes less precise, especially for the thresholding method in which precision depends directly on the sampling rate $F_{e}$.

In the experiments presented in this section, the M${}^{2}$APix sensor was fixed while a textured pattern was moving in front of it in a wide OF range and under controlled wide-range light levels. However, real flight conditions can generate disturbances due to vibrations or uncontrolled lighting conditions, which can change abruptly during the flight. Therefore, to test the two algorithms in real flight conditions, one M${}^{2}$APix sensor was mounted underneath a 350-gram quadrotor pointing downwards to measure the OF produced by the visual motion of the ground below.

## 5. Measuring Optical Flow in Flight

### 5.1. Method

Here, we evaluate the two algorithms running on the M${}^{2}$APix sensor under real flight conditions, in the presence of vibrations, movement disturbances (due to the MAV’s attitude stabilization) and light variations. To this end, the M${}^{2}$APix sensor was attached to the bottom of a 350-gram X4-MaG quadrotor [42], aligned to the direction of frontal movement of the MAV. In this configuration, OF measurements were only influenced by the linear speed vector’s components of the quadrotor and its pitch rotation movements. Light intensity was measured with a photodiode having the same spectral sensitivity, also attached to the bottom of the X4-MaG and oriented in the same downward direction as the M${}^{2}$APix sensor. To get the OF ground-truth with high accuracy, the X4-MaG flew inside the Mediterranean Flying Arena (http://www.flying-arena.eu) equipped with 17 motion-capture cameras covering a 6 × 8 × 6 m volume. The X4-MaG followed a predefined 3D trajectory using the VICON™ (Oxford, UK) system outputs as feedback signals.

**Figure 7:**
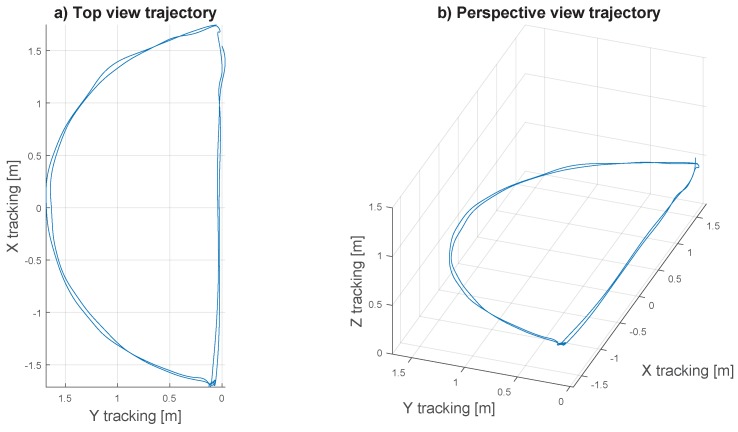
MAV trajectory: (**a**) top view; (**b**) perspective view. The two laps were performed at a flight height of 0.4 m.

The trajectory pattern retained for this experiment was a half-moon shape, positioned at a constant height of 0.4 m above the textured ground (cf. Figure 7). The X4-MaG was controlled to move along the half-circle trajectory segment at a constant speed of 0.5 m·s${}^{-1}$. During the straight trajectory segment, velocity first increased progressively from 0 to 1.5 m·s${}^{-1}$ and then decreased again to 0 m·s${}^{-1}$. Between these two segments, the X4-MaG hovered in place while making a pure yaw rotation. Since the speed vector of the X4-MaG is tangential to its trajectory, the half-moon pattern confronted the M${}^{2}$APix sensor with three distinct types of movement: translation, rotation and a combination of both.

**Figure 8:**
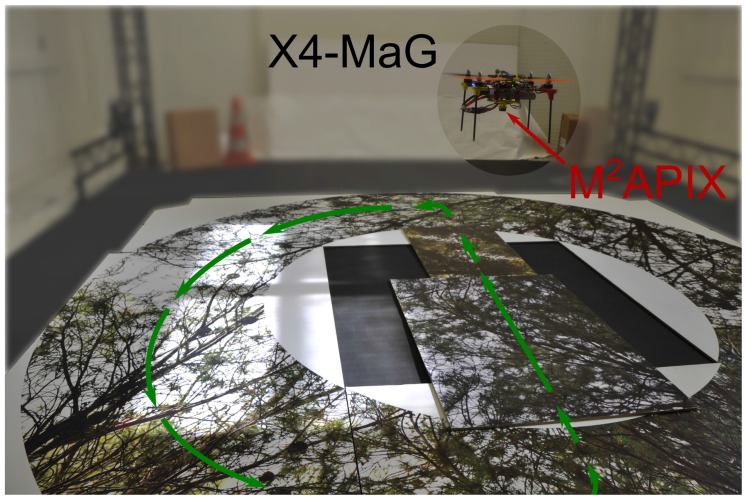
X4-MaG drone hovering at 1.2 m above the natural texture in the flight arena. The half-moon shaped trajectory is represented in green. The M${}^{2}$APix sensor is embedded under the quadrotor looking downwards and measuring the OF coming from the ground below. See Video S1 to watch the experiment.

Lighting was provided by three sources: daylight coming through the top windows, fluorescent tubes attached to the ceiling and infra-red light emitted by the motion capture system. Manipulation of the window blinds not only modulated the global intensity of the entering daylight, but also gave rise to high-range variations in lighting on the ground texture (see Figure 8). The intensity of the light emitted by the fluorescent tubes could be adjusted manually by means of a dimmer. Because it was necessary for the proper functioning of the tracking, the 9 $\times \;10^{-5}$ W· cm${}^{-2}$ irradiance provided by the motion capture system could not be altered.

### 5.2. In-Flight Results

**Figure 9:**
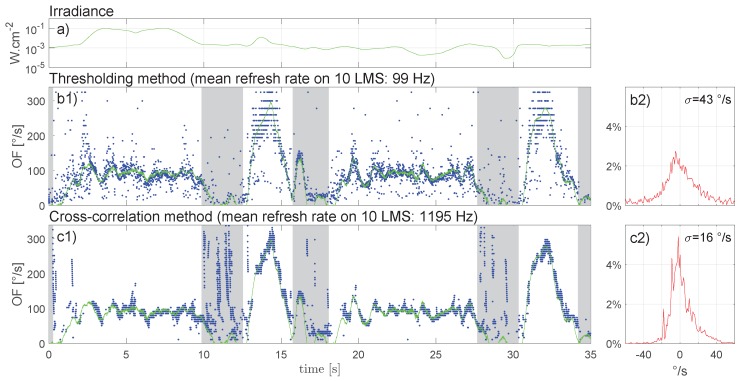
(**a**) Light intensity in W·cm${}^{-2}$ measured with a photodiode oriented in the same direction as the M${}^{2}$APix sensor embedded under the X4-MaG; (**b**,**c**) In the left panels (**b1**,**c1**), blue points represent single-LMS OF measurements; the green line is the ground-truth provided by high-precision Vicon tracking. Right panels (**b2**,**c2**) present the OF error distribution, with 1°/s beams in the [300°/s; 300°/s] range, with respect to the ground-truth during forward drone movement (white zones in left panels). The excluded (gray) zones correspond to periods of pure yaw rotation during the MAV trajectory. See Supplemental Movie 1 to watch the experiment.

Two flight laps of the pattern were recorded, during which the light was arbitrarily modulated. During the first lap, the blinds were left open, and daylight projected very luminous bands on the textured ground (Figure 8) that the drone encountered between the third and eighth seconds of the experiment (Figure 9a). As a result, irradiance during this first lap varied between $10^{-3}$ W·cm${}^{-2}$ and $10^{-1}$ W·cm${}^{-2}$. The blinds were then fully closed (at 10 s in Figure 9). Manual modulation of the fluorescent tubes induced irradiance variations ranging from 9$\times 10^{-5}$ W·cm${}^{-2}$ to 2 $\times \;10^{-3}$ W·cm${}^{-2}$.

In Figure 9b1,c1, white zones correspond to periods of forward movement of the drone, whereas gray zones correspond to hovering periods with 90° yaw rotation, orienting the drone in preparation of the next trajectory segment. Half-circle segments are identified by larger white zones between 0 and 10 s and between 18 and 28 s, and straight segments are identified by smaller white zones between 13 and 16 s and between 31 and 34 s. Thus, one large white zone and one small white zone represent one lap. For a trajectory height of 0.4 m and a speed range between 0.5 and 1.5 m·s${}^{-1}$ without pitch rotations, the OF theoretically varies from 72 to 215 °/s.

The OF oscillations observed during the half-circle part are mainly due to the drone’s attitude stabilization system, with the resulting pitch rotations inducing changes in OF.

Inspection of Figure 9b revealed that the thresholding method provided a virtually uninterrupted stream of OF measurements. Even in the presence of forward drone movement, measurements however showed quite some variability, as captured by the overall (excluding gray areas) standard deviation (*σ*) of 43 °/s. During forward movement (white zones), the OF-output refresh rate reached an average of 99 Hz for the 10 LMSs. The cross-correlation method (see Figure 9c) occasionally gave rise to measurement gaps, especially during the high acceleration phases (see Figure 9c at 13 s). However, compared to the thresholding method, measurement variability was lower with σ=16 °/s, and the OF-output refresh rate was much higher, reaching an average of 1195 Hz for the full M${}^{2}$APix sensor (10 LMSs).

The foregoing results bring out the differential capabilities of the two methods in different situations. Although, overall, the cross-correlation method provides better results in terms of precision, robustness, working range and output refresh rate, its use in MAV applications may be compromised by a crippling CPU load. Indeed, as demonstrated in Section 4 , for a single M${}^{2}$APix sensor, the cross-correlation method, operating at $F_{e}$ = 500 Hz, gave rise to an average CPU load of 52.5%, whereas with the thresholding method operating at $F_{e}$ = 1000 Hz, the CPU load was always lower than 3%. We therefore explored OF-output under reduced working frequencies, allowing lessening of the CPU load.

### 5.3. Offline Results at a Low Sampling Rate

**Figure 10:**
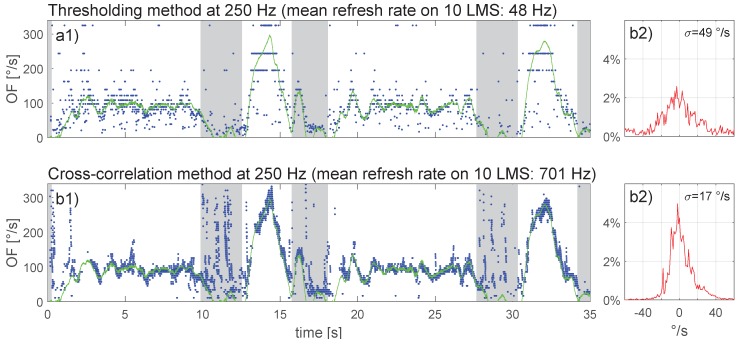
OF computed with (**a**) the thresholding method and (**b**) the cross-correlation method after down-sampling the data underlying Figure 9 to $F_{e}$ = 250 Hz.

**Figure 11:**
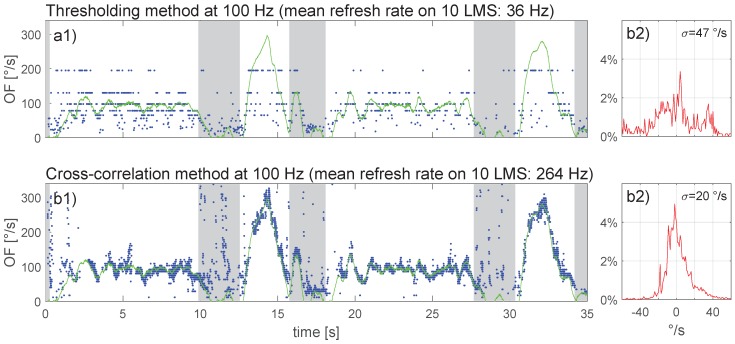
OF computed with (**a**) the thresholding method and (**b**) the cross-correlation method after down-sampling the data underlying Figure 9 to $F_{e}$ = 100 Hz.

The recorded flight data of the online calculations presented in Figure 9 were down-sampled before running the two algorithms again; the results of this offline OF computation are presented in the same format in Figure 10 with $F_{e}$ = 250 Hz and in Figure 11 with $F_{e}$ = 100 Hz.

Comparison of Figure 9 to Figure 11 (see also Table 1) revealed that for the thresholding method, a reduction of the sampling rate resulted in a stronger OF quantization (especially visible with higher OF values), accompanied by a decrease in OF-output refresh rate (from 99 Hz at $F_{e}$ = 1000 Hz to 36 Hz at $F_{e}$ = 100 Hz) and a widening of the error distribution. For the cross-correlation method, a reduction in sampling rate did not affect the error distribution to any noticeable extent, whereas the OF-output refresh rate decreased proportionally with the sampling rate (from 1195 Hz at $F_{e}$ = 500 Hz to 264 Hz at $F_{e}$ = 100 Hz).

## 6. Discussion and Conclusions

Experiments were performed to test two time-of-travel algorithms with an auto-adaptive bio-inspired silicon retina, called M${}^{2}$APix, intended for MAV applications. The M${}^{2}$APix sensor is a prototype sensor [12] composed of only 12 auto-adaptive pixels forming 10 LMSs. The auto-adaptive pixels allowed us to evaluate each time-of-travel algorithm largely independently of lighting conditions. In the field of robotics, working in a seven-decade range of irradiance is a considerable advantage because robots may then be led to work in environments subject to strong lighting variations, such as obstacle forests, urban canyons or inside buildings.

In the experiments underlying Figure 5, we controlled the lighting in a five-decade range of irradiance. The lower limit was reached at around $10^{-6}$ W·cm${}^{-2}$ irradiance, when the M${}^{2}$APix sensor was no longer able to deliver visual signals.

As can be seen in Figure 6, our results revealed that both time-of-travel algorithms are accurate at all OF speeds tested. However, the precision of the cross-correlation method ($C_{V}<0.1$ in Figure 6b) is considerably better than the thresholding method ($C_{V}>0.1$ in Figure 6a) over a wide OF range. At the lowest OF (25${}^{\circ }$/s), both methods strongly lack precision, probably due to the auto-adaption: working at a 0.5-s time constant, the auto-adaptive process may affect OF measurement at low speed when a time delay Δt approaches 0.5 s. It is in fact difficult to measure both slow and fast OFs with a constant inter-pixel angle Δφ; with $\Delta \varphi =1.5^{\circ }$, the thresholding allowed measuring an OF range of [1.5${}^{\circ }$/s; 25${}^{\circ }$/s] in real flight conditions [29]. Modulating Δφ as a function of the OF required appears to be the only way to measure the OF in the three-decade range (e.g., [1${}^{\circ }$/s; 1000${}^{\circ }$/s]) with a time-of-travel algorithm.

Experiments with a moving pattern and in real flight showed that even under varying lighting conditions, both time-of-travel algorithms are quite accurate with respect to ground-truth OF. The cross-correlation method however appears to be more precise with a higher output refresh rate than the thresholding method. For MAV applications with fully-embedded computational resources, the cross-correlation method requires 18-times more computational resources than the thresholding method. The cross-correlation was therefore tested offline at a low sampling rate (250 Hz in Figure 10b1, 100 Hz in Figure 11b1) and found to work well despite some brief measurement gaps when OF accelerated beyond 260${}^{\circ }$/s${}^{2}$ (e.g., Figure 10b1). The thresholding method, on the other hand, only worked adequately at a high sampling rate (1 kHz in Figure 9b1); results were poor at a low sampling rate (250 Hz in Figure 10a1 or 100 Hz in Figure 11a1). For future work, it appears worthwhile to explore combinations of both methods by merging their OF measurements (e.g., a cross-correlation method running at 250 Hz with a thresholding method working in parallel and running at 1 kHz), as this would allow profiting from the benefits of each method without too strongly loading the CPU.

Results presented in Table 1 will allow future research to choose between the time-of-travel algorithms for measuring the OF as a function of embedded computational resources. If an algorithm using little computational resources is required (e.g., for computing dozens or hundreds of LMSs, while using one and the same embedded target), the thresholding method may be preferred. By adding a median filter eliminating matching errors [8], the thresholding method moreover becomes more precise. Its precision can also be improved when running at 200 Hz $\leq F_{e}$ < 1 kHz using a linear interpolation applied to the photoreceptor signals [30]. While the cross-correlation method is inherently both accurate and precise and can provide a high output refresh rate (that is, about proportional to the sampling rate $F_{e}$), it requires much more computational resources (also approximately proportional to the sampling rate $F_{e}$), which will limit the number of LMSs that can be embedded into the same target. The CPU load of the cross-correlation method may be reduced by using on-board digital signal processing (DSP).

Finally, from a robotics point of view, our 350-gram X4-MaG quadrotor was equipped with an IMU working at 250 Hz to stabilize attitude and with a downward OF sensor, composed of 10 LMSs running at a refresh output frequency close to the IMU sampling rate. A high refresh rate for both inertial and visual sensors will be a perquisite in the near future to endow MAVs with strong reactive abilities in unpredictable environments. Moreover, this study has demonstrated that a time-of-travel algorithm coupled with a M${}^{2}$APix sensor can accurately measure the OF in a wide range [25°/s; 1000°/s], allowing MAVs to fly close to surrounding obstacles at high speed in any lighting conditions.

## Figures and Tables

**Table 1:** Comparison between the results obtained with the two time-of-travel algorithms.

	Thresholding Method				Cross-Correlation Method			
**Sampling Rate ($F_{e}$)**	**1 kHz**	**500 Hz**	**250 Hz**	**100 Hz**	**1 kHz**	**500 Hz**	**250 Hz**	**100 Hz**
CPU Load (%)	2.2	1.1 *	<1 *	<1 *	overload	52.5	26.3 *	10.5 *
Precision *σ* (°/s)	43	44	49	47	-	16	17	20
Refresh rate (Hz/10 LMSs)	99	51	48	36	-	1195	701	264

* Theoretical value because corresponding tests were made offline.

## Supplementary Materials

The following are available online at http://www.mdpi.com/1424-8220/17/3/571/s1, Video S1: a video of the experiment with the micro flying robot.

## Abbreviations

The following abbreviations are used in this manuscript:

OF	Optical flow
LMS	Local motion sensor
M${}^{2}$APix	Michaelis–Menten auto-adaptive pixel
